# Early Detection of Mobility Decline: Smartphone-Based Analysis of Gait and Stair Negotiation

**DOI:** 10.1093/geroni/igaf122.789

**Published:** 2025-12-31

**Authors:** Roee Hayek, Rebecca Brown, Itai Gutman, Guy Baranes, Shmuel Springer

**Affiliations:** Ariel University, Ariel, HaMerkaz, Israel; University of Pennsylvania, Philadelphia, Pennsylvania, United States; Ariel University, Ariel, HaMerkaz, Israel; Ariel University, Ariel, HaMerkaz, Israel; Ariel University, Ariel, HaMerkaz, Israel

## Abstract

Aging is associated with gradual mobility decline, often undetected until it affects daily activities. Although most mobility impairments are linked to old age, they can emerge as early as middle age. We examined the utility of smartphone-based accelerometry to detect early age-related mobility changes. Participants were 88 healthy adults divided into four age groups, young (20-35 years), early middle-aged (45-54 years), late middle-aged (55-65 years), and older adults (65-80 years). They completed gait assessments with and without cognitive (texting) and physical (carrying load) dual tasks and stair negotiation test. Gait measures included gait speed (m/sec), stride time variability (%), and movement similarity, measured with dynamic time warping (DTW) of anteroposterior (AP) and mediolateral (ML) acceleration signal. Dual-task cost (DTC) of the cognitive and physical tasks were calculated for each gait measure. Stair negotiation outcomes included total ascent and descent time (sec), muscle power (watts/m), and movement similarity using vertical (V) DTW. Late middle-aged adults had significantly higher cognitive DTC of stride time variability than young adults (p = 0.006). A strong indicator of early mobility decline was movement similarity. Young adults showed significantly lower cognitive DTC of DTW-AP compared to all other groups, early middle-age (p = 0.035), late middle-age (p = 0.007) and older adults (p = 0.006), as well as lower DTW-V during stair ascending (p < 0.001, compared to all groups). These findings highlight the utility of smartphone-based indices, particularly movement similarity, to detect subtle mobility changes in midlife, allowing for targeted interventions to promote healthy aging.

